# Functional Expression of *Gloeobacter* Rhodopsin in PSI-Less *Synechocystis* sp. PCC6803

**DOI:** 10.3389/fbioe.2019.00067

**Published:** 2019-03-29

**Authors:** Que Chen, Jos Arents, J. Merijn Schuurmans, Srividya Ganapathy, Willem J. de Grip, Otilia Cheregi, Christiane Funk, Filipe Branco dos Santos, Klaas J. Hellingwerf

**Affiliations:** ^1^Molecular Microbial Physiology Group, Swammerdam Institute for Life Sciences, University of Amsterdam, Amsterdam, Netherlands; ^2^Center of Synthetic Biochemistry, Institute of Synthetic Biology, Shenzhen Institutes of Advanced Technology, Chinese Academy of Sciences, Shenzhen, China; ^3^Aquatic Microbiology, Institute for Biodiversity and Ecosystem Dynamics, University of Amsterdam, Amsterdam, Netherlands; ^4^Biophysical Organic Chemistry, Leiden Institute of Chemistry, Leiden University, Leiden, Netherlands; ^5^Department of Chemistry, Umeå University, Umeå, Sweden

**Keywords:** retinal-based proton pump, PSI-deletion *Synechocystis*, growth stimulation, carotenoid metabolism, oxygen evolution

## Abstract

The approach of providing an oxygenic photosynthetic organism with a cyclic electron transfer system, i.e., a far-red light-driven proton pump, is widely proposed to maximize photosynthetic efficiency via expanding the absorption spectrum of photosynthetically active radiation. As a first step in this approach, *Gloeobacter* rhodopsin was expressed in a PSI-deletion strain of *Synechocystis* sp. PCC6803. Functional expression of *Gloeobacter* rhodopsin, in contrast to Proteorhodopsin, did not stimulate the rate of photoheterotrophic growth of this *Synechocystis* strain, analyzed with growth rate measurements and competition experiments. Nevertheless, analysis of oxygen uptake and—production rates of the *Gloeobacter* rhodopsin-expressing strains, relative to the ΔPSI control strain, confirm that the proton-pumping *Gloeobacter* rhodopsin provides the cells with additional capacity to generate proton motive force. Significantly, expression of the *Gloeobacter* rhodopsin did modulate levels of pigment formation in the transgenic strain.

## Introduction

Human society faces a growing tension between the increasing use of fossil, i.e., petroleum-based, fuel and the wish to decrease the alarming level of CO_2_ emission. Thus, developing methods for the sustainable production of fuel and chemical commodities for materials synthesis, has become one of the most imperative challenges of this century. Solar energy is considered as the most abundant and most suitable form of sustainable energy available on the earth's surface. Converting solar energy with the highest possible efficiency is therefore an important topic for both the basic- and the applied sciences.

A widely acclaimed proposal to achieve this is to expand the spectrum of photosynthetically active radiation (PAR) for phototrophic microorganisms. For oxygenic photosynthetic microorganisms this PAR region is almost exclusively limited to photons with a wavelength ≤ 700 nm (Kühl et al., 2005; Zhu et al., 2008; Chen et al., 2010). These are about half of the total number of photons available from the sun, that reach the surface of the earth (Ooms et al., 2016). A more moderate expansion, e.g., up to 750 nm, would already increase the number of available photons for oxygenic photosynthesis with around 19% (Chen and Blankenship, 2011).

Notably, solar energy conversion systems naturally exist that do function with light of wavelengths >700 nm, i.e., those oxygenic photosynthetic microorganisms that use chlorophyll *d* (Chl *d*) (Manning and Strain, 1943; Von Wettstein et al., 1995) or chlorophyll *f* (Chl *f*) (Chen et al., 2010; Li et al., 2012; Ho et al., 2016), instead of chlorophyll *a*. These two alternative chlorophylls capture photons in the range of 700–720 and 700–740 nm, respectively. Many additional examples can be found in bacteria that carry out anoxygenic photosynthesis. In this process the energy of photons of much longer wavelength, i.e., up to 1,100 nm (Brock et al., 2011), is sufficient to initiate the primary reactions of photosynthesis. Thus, introducing a heterologous infra-red absorbing photosystem, like a cyclic electron transfer system of an anoxyphototroph (Blankenship et al., 2011; Ort et al., 2015), may be a straightforward approach for the exploitation of the full spectrum of solar radiation. In a more modest approach it was recently accomplished to heterologously express Chl *f* in the cyanobacterium *Synechococcus* 7,002. However, the low level of chlorophyll production that was achieved presumably prevented phenotypic effects of this approach. Heterologous expression of all the components required for functional expression of a cyclic electron transfer chain indeed is challenging. As an alternative, expression of a far-red-shifted retinal-based proton pump (Chen et al., 2016a,b; Ganapathy et al., 2017) could be an option, as we demonstrated previously via expression of an engineered, red-shifted, retinal-based proton pump in a mutant strain of *Synechocystis* sp. PCC6803, impaired in retinal synthesis (Chen et al., 2018b).

*Gloeobacter* rhodopsin (GR) was identified in a primitive cyanobacterium, *Gloeobacter violaceus* PCC7421 (Rippka et al., 1974). *In vitro* studies have shown that this protein has a 2-fold faster turnover rate than Proteorhodopsin (Wang et al., 2003; Miranda et al., 2009; Chen et al., 2017; Ganapathy et al., 2017). More interestingly, it is able to bind carotenoids with a 4-keto group, e.g., salinixanthin and echinenone, to increase the absorption cross-section of the pump (Luecke et al., 2008; Imasheva et al., 2009; Balashov et al., 2010). Proteorhodopsin expression significantly enhances the growth rate of both wild type *Synechocystis* and its PSI-deletion (ΔPSI) derivative, when grown in a batch culture under 25 μmol · m^−2^ · s^−1^ green light (Chen et al., 2018a). To further increase the energy contribution from retinal based phototrophy, and to compare which of the two available proton pumps (i.e., Proteorhodopsin and *Gloeobacter* rhodopsin) has the higher efficacy in this type of energy conversion, we next expressed GR in a ΔPSI strain of *Synechocystis*. However, unlike Proteorhodopsin (Chen et al., 2018a), *Gloeobacter* rhodopsin did not significantly increase the growth rate of this *Synechocystis* strain. Nevertheless, analysis of oxygen uptake and—evolution rates of the *Gloeobacter* rhodopsin-expressing strains demonstrate that, relative to the ΔPSI control strain, the proton-pumping *Gloeobacter* rhodopsin provides the cells with additional capacity to generate proton motive force. In addition, spectroscopic analysis shows that the *Gloeobacter* rhodopsin-expressing strain has a significantly altered absorption profile, suggesting that biosynthesis of photosynthetic pigments has been modulated in this strain.

## Materials and Methods

### Strains and Growth Conditions

Strains of *Escherichia coli* were routinely grown in LB-Lennox (LB) liquid medium at 37°C with shaking at 200 rpm, or on solid LB plates containing 1.5% (w/v) agar.

The ΔPSI-derivative of *Synechocystis* sp. PCC 6803 (a glucose tolerant strain; Shen et al., 1993; Hernandez-Prieto et al., 2011) was routinely grown at 30°C with continuous illumination with red, green and blue light (RGB-light) at a total light intensity of 28.3 μmol · m^−2^ · s^−1^ (containing 3 μmol · m^−2^ · s^−1^ red, 25 μmol · m^−2^ · s^−1^ green, and 0.3 μmol · m^−2^ · s^−1^ blue light). The red, green and blue LEDs emitted maximally at 635, 527, and 459 nm, respectively. Liquid cultures were grown in BG-11 medium (Sigma Aldrich), supplemented with 10 mM glucose, 50 mM Piperazine-N,N′-*bis*(3-propanesulfonic acid) (Pipps) (pH 8.0) and appropriate antibiotics, and with shaking at 120 rpm (Innova 43, New Brunswick Scientific). The BG-11 agar plates were supplemented with 25 mM Pipps (pH 8), 10 mM glucose, 0.3% (w/v) sodium thiosulfate, and 1.5% (w/v) agar.

Where appropriate, antibiotics were added to a final concentration of: kanamycin (25–50 μg/ml) and chloramphenicol (35 μg/ml), either separately or in combination.

### Conjugation

Plasmids were transferred to the ΔPSI *Synechocystis* strain via tri-parental mating, essentially as described before (Chen et al., 2016b). These plasmids included pQC006 (for expression of His-PR, Chen et al., 2016b), pQC012 (for expression of His-GR; Chen et al., 2017); and plasmid pJBS1312 (empty-plasmid control; Chen et al., 2016b). The presence of the plasmids was confirmed with appropriate PCR tests, carried out after the conjugation procedure. Strains or plasmids used in this study are summarized in Table 1.

**Table 1:** Strains or plasmids constructed for this study.

**Strain or plasmid**	**Relevant characteristics^a^**	**Source or references**
**STRAINS**		
***Synechocystis*** **sp. PCC6803**		
ΔPSI	Cam^R^; Δ*psaAB*:: ${\text{C}}_{\text{m}}^{\text{R}}$; a PSI deletion strain derived from glucose tolerant *Synechocystis sp*. PCC6803	Shen et al., 1993
QC-0	Δ*psaAB* (pJB1312); Cam^R^; kan^R^; an “empty plasmid” carrying ΔPSI *Synechocystis*	Chen et al., 2018a
QC-PR	Δ*psaAB* (pQC006); Cam^R^; kan^R^; a 6 × histine tagged PR-expressing ΔPSI *Synechocystis*	Chen et al., 2018a
QC-GR	Δ*psaAB* (pQC012); Cam^R^; kan^R^; a 6 × histine tagged GR-expressing ΔPSI *Synechocystis*	This study
***Escherichia coli***		
XL1-Blue	Cloning host	Agilent technologies
J53/RP4	Helper strain	Bachmann, 1972; Jacob and Grinter, 1975
**PLASMIDS**		
pJBS1312	kan^R^; expression vector, pVZ321 origin, P*_*psbA*2_*	Chen et al., 2016b
pQC012	kan ^R^; pJBS1312-based expression of GR, C-terminal 6 × histine tagged	Chen et al., 2016b

a *Ω is short for the omega resistance cassette; Cm^R^ represents the chloramphenicol resistance; while Amp^R^ means the ampicillin resistance cassette; Kan^R^ for kanamycin resistance; Spc^R^ for spectinomycin resistance and Str^R^ for streptomycin resistance*.

### Effect of Expression of a Bacterial Rhodopsin on Photo-Mixotrophic Growth of the ΔPSI *Synechocystis* Strain

Cells were grown in commercial BG-11 medium, supplemented with 10 mM glucose at 30°C with illumination with 28.3 μmol · m^−2^ · s^−1^ RGB light (see: section Strains and Growth Conditions). Growth of two strains was analyzed in parallel: ΔPSI *Synechocystis* strains expressing GR-His (QC-GR), and the “empty” plasmid (QC-O), respectively. An identical number of cells of each strain were harvested and washed, then inoculated into three 10-ml cultures for each strain in triplicate. Growth was monitored, essentially as described in Chen et al. (2018a), via cell density by measuring the OD_730_ of a small volume (150 μl), as well as via the number of cells per ml of sample.

### Growth Competition

The competition experiment and analysis of the growth-competition experiments with PCR were carried out essentially as described in Chen et al. (2018a). In short, an identical amount of cells from two strains: QC-0 and QC-GR were mixed together and inoculated into an Erlenmeyer with 10 ml medium. The experiment was carried out in BG-11 medium supplemented with 10 mM glucose, at an intensity of incident illumination of 28 μmol · m^−2^ · s^−1^ RGB light. During the course of the experiment, an aliquot of this culture was diluted to OD_730_ = 0.1 in an Erlenmeyer with 10 ml fresh medium every 2 days, in order to maintain exponential growth. One milliliter culture was removed every day for measurement of cell density and estimation of the abundance of the GR gene by PCR. The final culture, obtained after 16 days, was diluted 1,000 times, after which 10 μl of the diluted culture were plated on a BG-11 plate (supplemented with 10 mM glucose). The single colonies emerging were used to determine the number of colony forming units (CFU) of each strain, by colony PCR. Each experiment was carried out with three biological replicates.

### Analysis of the Growth-Competition Experiments With PCR

Equal numbers of cells of the collected samples were added into a PCR reaction mixture as the template, and primers JBS315/JBS316, which specifically bind to the plasmid backbone, rather than to the part actually expressing the GR, were used for PCR amplification. The amplified product from the “empty plasmid” (pJBS1312) and from the GR-His encoding plasmid (pQC012), have a size of 578 and 1,493 bps, respectively. The intensity of each band was measured using ImageJ v1.49a (W.S. Rasband, U.S. National Institutes of Health, http://imagej.nih.gov/ij/). The ratio in band intensities of two bands derived from the same sample was calculated, and compared with a standard curve formed by the ratio in band intensities of two bands plotted against a series of known ratios of mixed cells between QC-GR and QC-0. Standards were prepared by mixing cells of the two strains (i.e., QC-GR and QC-0) in a series of known ratios, varying from 0/100 to 100/0 of GR-expressing cells over “empty plasmid” containing cells, in 10 steps of 10%. The detailed PCR protocol was carried out, essentially as described in (Chen et al., 2018a).

### Measurement of Glucose Content in Spent Medium

The samples for analysis of glucose content were collected from the three cultures that were used to compare the rate of photomixotrophic growth. Concurrently with the OD measurements, 100 μl of each culture was harvested for quantification of the residual glucose content of the medium. Glucose content in the resulting supernatant was determined using the D-Fructose/D-Glucose Assay Kit (Megazyme, U.S.A), in 96-well plates with a microplate photometer.

The rate of glucose consumption was calculated in terms of micromoles of glucose consumed per 10^9^ cells per hour. As the dynamics of glucose consumption and cell proliferation change along with the residual glucose content, we calculated the glucose consumption rate for each specific time window (i.e., between t_n−1_ and t_n_; t corresponds to the time of measurement, n refers to the number of measurements). In each time window, the amount of consumed glucose was calculated from the decrease in glucose content per ml culture, while the number of cells was taken as the average number of cells per ml measured at t_n−1_ and t_n_.

### Retinal Quantification

Retinal content was measured as a probe for quantification of *holo*-GR-His expression *in vivo*, in cell pellets that were collected from a shake flask culture in the exponential growth phase. The protocol was carried out, essentially as described in Chen et al. (2016b). In short, all-*trans* retinal was converted into the more stable compound retinal oxime (Groenendijk et al., 1979), via reacting with 1 M hydroxylamine. The resulting reaction mixtures were subsequently extracted at least three times with petroleum ether (boiling point 40–60°C) and finally dissolved in n-heptane (HPLC grade). The extracted pigments were separated on an HPLC system with a C18 column (EC 150/4.6 NUCLEOSIL 100-5, MACHEREY-NAGEL), and n-heptane (HPLC grade) at 1 ml · min^−1^ as the mobile phase.

Elution of retinal oxime was monitored at 354 nm in our system. To determine the retinal content in a sample, the peak area of retinal oxime in the sample was compared with that of a series of known amounts of standards (all-*trans*-retinal (oxime), purchased from Sigma-Aldrich).

### Membrane-Inlet Mass Spectrometry

Rates of net oxygen production and oxygen consumption were measured by Membrane-inlet mass spectrometry (MIMS), essentially as described in Schuurmans et al. (2015) and Chen et al. (2018a), via the concentration of oxygen isotopes ^32^O_2_ and ^36^O_2_, respectively. In short, MIMS measurements were performed in a 10 ml air-tight cuvette containing a PSI-deletion *Synechocystis* culture, at a cell density (OD_730_) of 1.0. Prior to the experiment, the culture was dark adapted for 30 min and then briefly (~10 s) flushed with N_2_ to reduce the prevalent O_2_ concentration. Subsequently, the cuvette was closed and ^36^O_2_ was added into the reaction cuvette. The samples were subjected to orange light (634 nm) at intensities ranging from 0 to 200 μmol · m^−2^ · s^−1^, or green light (532 nm) at intensities ranging from 0 to 200 μmol · m^−2^ · s^−1^. Cells were subjected first to the low light intensity of 5 μmol · m^−2^ · s^−1^ for 10 min, to assure light adaptation, and to all subsequent light intensities for 3 min. After testing the different light intensities, dark respiration was measured during the next period of 3 min. At the timescale of these experiments corrections for a decrease in the oxygen concentration, due to oxygen “consumption” by the mass spectrometer, was not necessary.

### UV/Vis Absorption Spectroscopy

Cells of the strain of interest were harvested, washed three times with, and then re-suspended in, fresh BG-11 medium supplemented with 10 mM glucose. Absorption spectra of intact cells were measured with a SPECORD® 210 PLUS spectrophotometer (Analytik Jena, Germany), to minimize the contribution of light scattering. The spectra were corrected based on their light scattering in the range of 730−850 nm, and normalized on the phycobilin absorbance band at 625 nm.

## Results and Discussion

### GR Expression Did Not Stimulate the Photomixtrophic Growth of a ΔPSI *Synechocystis* Strain

**Figure 1:**
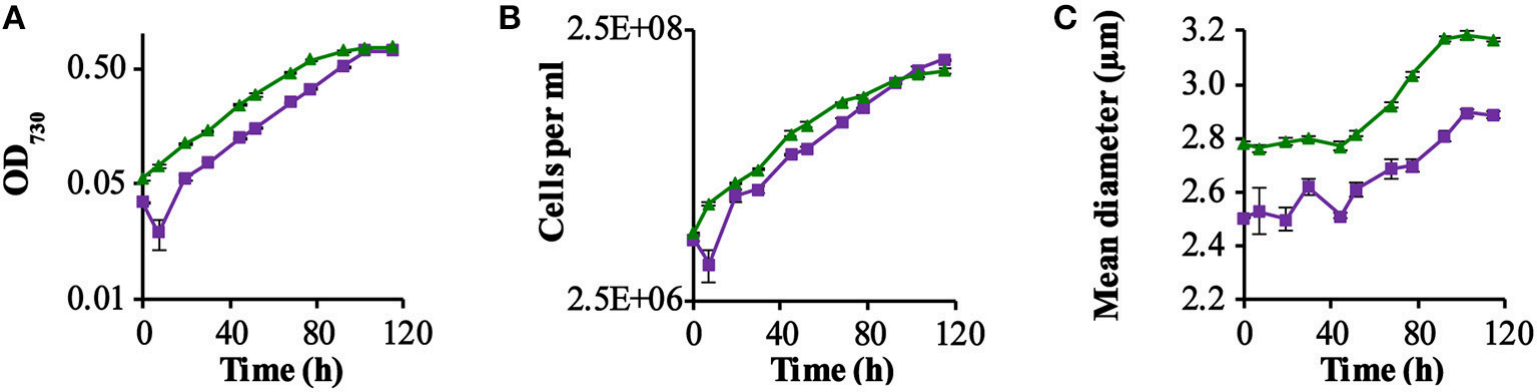
GR-stimulated growth in ΔPSI *Synechocsytis* strains. Growth comparison among the two strains QC-0 (Green triangles) and QC-GR (Purple squares), with illumination with 28 μE RGB light. All strains were grown in BG-11 medium supplemented with 10 mM glucose. Error bars represent the standard deviation of biological replicates within the experiment (*n* = 3), and are only visible when they exceed the size of the symbols. Time point zero indicates the time of inoculation of the cultures from a pre-culture growing linearly in the same medium. The growth was monitored via cell density at 730 nm (OD_730_; **A**) in a SPECTROstar Nano Microplate Photometer, number of cells per ml **(B)**; and mean diameter **(C)** as measured with a Casy Counter.

We compared—in shake flask cultures—the growth rate of two strains: a strain expressing GR (QC-GR) and the control strain containing the “empty” plasmid (QC-0). The experiment was carried out in commercial BG-11 medium, additionally containing 10 mM glucose, by inoculating the same number of cells of each strain in 10 ml medium. We deliberately supplied an incident light intensity of low-intensity red and blue light (combined intensity ~3 μmol · m^−2^ · s^−1^), as the ΔPSI strain is sensitive to inhibition by relatively high light intensities (Shen et al., 1993), in combination with relatively intense green light (~25 μmol · m^−2^ · s^−1^). The green light we supplied (λ_max_ = 527 nm with a bandwidth of 34 nm) can strongly activate proton pumping in GR (Ganapathy et al., 2017) but is poorly absorbed by the photosynthetic apparatus of cyanobacteria (Chen and Blankenship, 2011). Growth was monitored via the optical density of the culture and via the number of cells per ml (Figure 1).

As Figure 1 shows, the GR expressing strain QC-GR grew significantly slower than the control strain QC-0, until around 95.2 h. After that, QC-GR overtook QC-0 in growth and generated a higher cell yield than the QC-0 strain (1.95 × 10^8^ and 1.56 × 10^8^ cells per ml culture, respectively). Under these conditions, apparently, expressing GR seems to have no significant stimulatory effect on the growth rate of a PSI-less strain.

Beyond that, it was observed that cells of the control strain, QC-0, consistently maintained a larger cell size (i.e., diameter) than the GR-His expressing strain QC-GR, during all stages of growth (Figure 1C), which is consistent with the cell-size difference between strain QC-0 and QC-PR reported before (Chen et al., 2018a). The difference in cell size also explains why, at the zero-time point, the optical density (OD_730_, reflecting cell scattering) of the QC-0 strain was higher than that of the QC-GR strain, while both cultures contained the same number of cells. We do not have a clear explanation, however, for the cause of this difference in cell size between these two strains.

To straightforwardly measure the contribution to the growth rate of the ΔPSI strain, attributable to the expression of the *Gloeobacter* rhodopsin, a growth competition experiment was designed. This experiment was initiated with the same number of cells of each of the two competing strains (i.e., QC-GR and QC-0). Via sampling from this competition experiment, and by making use of a calibration curve and an optimized PCR protocol (Chen et al., 2018a; see section Materials and Methods), the percentage of each of the two cell types could be calculated from the relative amounts of the PCR products of the two strains. With the use of the optimized PCR protocol, standard curves of the abundance of the two strains were obtained that could be fitted well (*R*^2^ > 0.96) with a second order polynomial.

**Figure 2:**
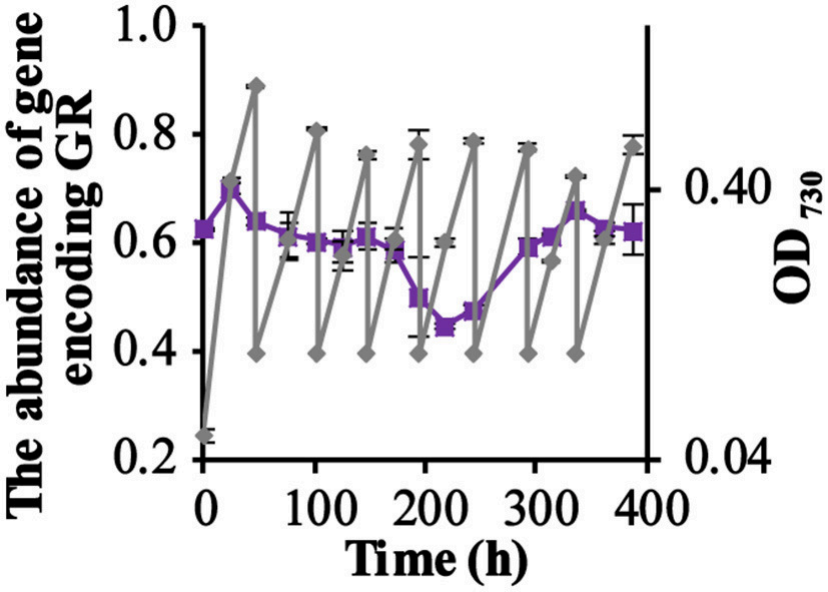
The competition for growth rate between the GR-expressing ΔPSI *Synechocystis* strain QC-GR and the QC-0 strain, under illumination with RGB light. The relative fraction of the gene encoding GR during the competition experiment under illumination with RGB light (Purple squares), quantitated using PCR. Growth of the mixture of the two strains was monitored via cell density at 730 nm (OD_730_, gray diamonds). Error bars represent the standard deviation of biological replicates within the experiment (*n* = 3), and are only visible when they exceed the size of the symbols. Time point zero indicates the time of inoculation of the cultures from a pre-culture growing linearly in the same medium.

Figure 2 shows that the fraction of GR expressing cells (QC-GR) continued to fluctuate around 60% through the entire experiment. PCR analysis of 120 single colonies, derived from samples taken on the last day (day 16), showed that 58% of the colonies (40 colonies per plate tested, with three biological replicates) carried the GR-encoding gene. These results fully confirm that expression of GR did not bring the ΔPSI-strain of *Synechocystis* a detectable stimulatory growth advantage under the conditions selected.

### Assay of the Residual Glucose Content in Spent Medium

**Figure 3:**
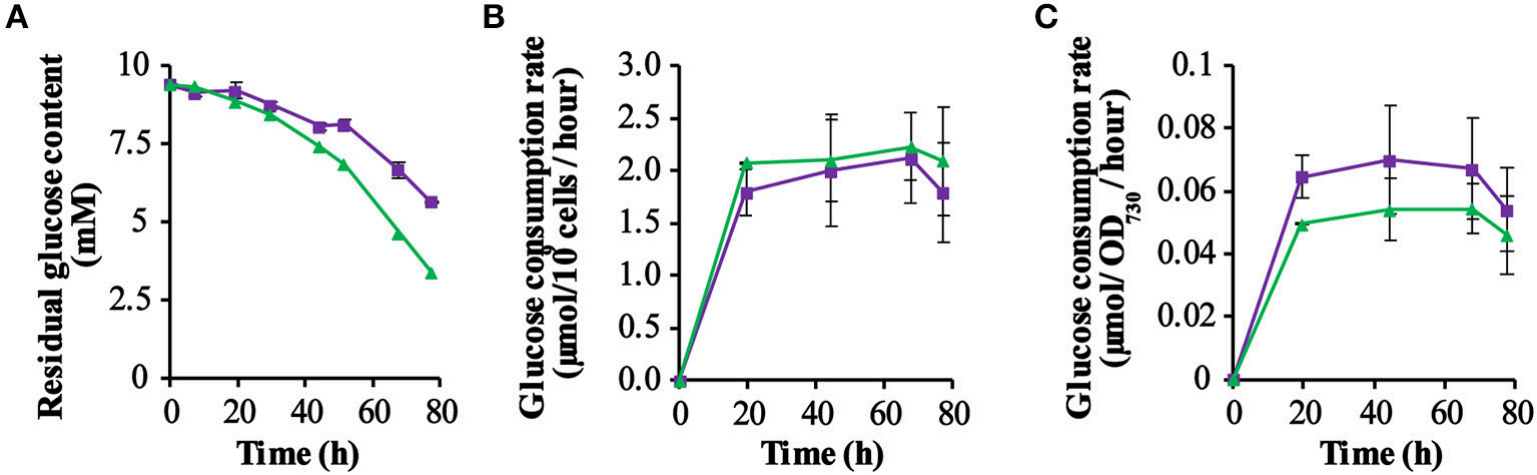
Kinetics of glucose consumption of the strains QC-0 (green triangles) and QC-GR (purple squares). The residual glucose concentration in the culture was measured using a glucose-content determination kit (for further detail: see section Materials and Methods). **(A)** Shows the dynamics of the remaining glucose concentration in the spent medium. **(B,C)** Show the glucose consumption per 10^9^ cells per hour, and the glucose consumption per OD_730_ per hour, respectively. Error bars represent the standard deviation of biological replicates within the experiment (*n* = 3), and are only visible when they exceed the size of the symbols. Time zero indicates inoculation of the cultures from a pre-culture growing linearly in the same medium.

As the PSI-deletion strain was grown photo-mixotrophically in a medium with 10 mM glucose, we decided to monitor the glucose consumption of each strain by measuring the residual extracellular glucose content during growth in a batch culture. From the results obtained (Figure 3A) it is clear that the glucose content decreased measurably faster in cultures of the control strain QC-O than in the GR expressing strain QC-GR, although after 92.5 h the glucose was exhausted in both cultures. However, this probably reflects the initially lower cell density of the QC-GR strain, since comparison of the cell-specific- (Figure 3B) and the biomass-specific (i.e., based on OD_730_; Figure 3C) glucose consumption rate shows that two strains have similar glucose consumption rates (i.e., ~2 μmol per 10^9^ cells per hour).

### GR Expression Did Provide Extra Proton Motive Force/ATP to a ΔPSI *Synechocystis* Strain

In order to establish whether expression of GR in the QC-GR strain does contribute to proton motive force generation, we set up MIMS measurements, which allow one to measure the change in the concentration of supplemented ^32^O_2_ and ^36^O_2_ as a function of time. If a reaction is initiated with only ${\text{H}}_{2}^{16}$O present, any increase in the concentration of ^32^O_2_ directly reflects the rate of oxygen evolution by PS-II. If simultaneously oxygen consumption takes place, the observed rate of oxygen production has to be increased with the simultaneous rate of oxygen consumption, in order to derive the actual rate of oxygen evolution. When ^36^O_2_ is supplied the rate of oxygen consumption can be derived from the rate of ^36^O_2_ oxygen consumption. The two oxygen isotopes are then present at e.g., equal concentrations, which should strongly exceed the Km's of the oxygen consuming enzymes (like respiratory oxidases, the Flv1/3 proteins, Rubisco, etc.).

**Figure 4:**
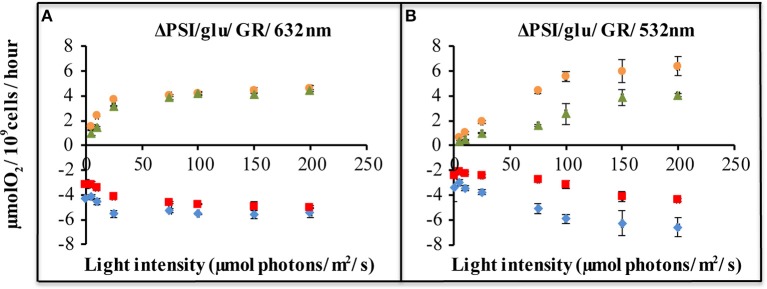
Light-driven net oxygen production and consumption, as measured with a membrane-inlet mass spectrometer (MIMS), of ΔPSI *Synechocystis*, with and without GR expression. Strains carrying plasmid pJBS1312 or plasmid pQC012 were compared for an analysis of the effect of GR-expression. Oxygen exchange was measured with MIMS under illumination with orange-red [632 nm, **(A)**], or green light [532 nm, **(B)**] at intensities varying from 0 to 200 μE. Prior to measurements, cells were washed and re-suspended in fresh BG-11 medium supplemented with 10 mM glucose and dark-adapted for 30 min. ΔPSI/glu: ΔPSI *Synechocystis* grown with addition of glucose. [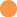]: O_2_ evolution of strains carrying “empty plasmid” pJBS1312; [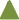]: O_2_ evolution of strains carrying plasmid pQC012 (GR-His); [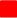]: O_2_ uptake of strains carrying plasmid pQC012 (GR-His). [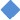]: O_2_ uptake of strains carrying “empty plasmid” pJBS1312; Error bars represent the standard deviation of biological replicates within the experiment (n ≥ 3), and are only visible when they exceed the size of the symbols.

Comparison of the rates of oxygen evolution and oxygen consumption, between the ΔPSI strain, with and without expression of *Gloeobacter* rhodopsin, shows that a significant difference only occurred upon illumination with green light (Figure 4B), and not with orange light (Figure 4A). The QC-GR strain had a significantly lower rate of oxygen consumption (up to 50% less, dependent on light intensity), as well as a lower oxygen evolution rate (up to 46% lower) than the control strain QC-0. This indicates that GR generates proton motive force over the thylakoid membrane and accordingly inhibits electron transfer driven by: (i) respiratory electron transfer to cytochrome c oxidase and (ii) PSII dependent oxygen evolution.

The decrease in the rates of oxygen evolution and—consumption, caused by expression of GR is at a comparable level to that cause by expression of PR. Evidently, expression of GR also provides the ΔPSI strain with an extra pathway for proton motive force generation and/or ATP synthesis, probably of a similar size as PR (Chen et al., 2018a). Measurements of all-*trans* retinal content indicate that, in the exponential phase, the QC-PR strain contains about 2.7 × 10^4^ molecules *holo*-PR per cell while the QC-GR strain carries about 1.3 × 10^4^ molecules *holo*-GR per cell. However, this lower *holo*-GR level may be compensated by its faster pumping rate, so that GR could contribute approximately to the same extent to extra proton motive force in the ΔPSI strain of *Synechocystis* as Proteorhodopsin.

### GR Expression Strongly Modulates Carotenoid Metabolism

**Figure 5:**
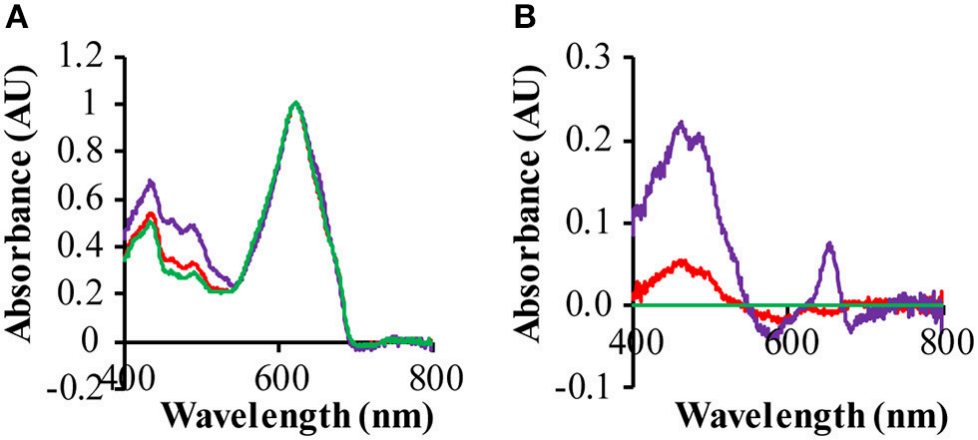
Effect of rhodopsin expression on the absorption spectra of the ΔPSI strains. Cells were grown under 3 μE red/blue and 25 μE green light in BG-11 medium, supplemented with 10 mM glucose. Prior to measurement, cells were washed with, and then re-suspended into, fresh BG-11 medium. **(A)** Absorption spectra of QC-0 (green), QC-PR (red) and QC- GR (purple) were baseline-corrected based on their light scattering in the range of 730−850 nm and then normalized on their absorption at 625 nm; **(B)** difference spectra of QC-PR and QC-GR against QC-0, taken from **(A)**.

The ΔPSI strain shows a significant decrease of its 680 and 440 nm absorption bands, because of the loss of a large fraction of its chlorophyll a, due to the deletion of the PSI core proteins. Hence in these strains the absolute spectra are dominated by the absorbance of the phycobilisomes, absorbing maximally at 625 nm. The difference spectrum of a GR-expressing strain (QC-GR) relative to a control strain (QC-0), reveals much higher absorption in the range of 400 to 550 nm, and a specific absorption peak at 652 nm in the GR-expressing strain (Figures 5A,B). This indicates a higher accumulation level of carotenoids and extra allophycocyanin, respectively. In combination with the fact that GR has the capacity to bind a keto-carotenoid as antenna chromophore (both echinenone and hydroxyl-echinenone can be bound Balashov et al., 2010; Chen et al., 2017; Jana et al., 2017, we propose here that GR may compete with, and take away, part of the keto-carotenoids from other keto-carotenoid binding proteins in *Synechocystis*, which then may lead to the overexpression of other carotenoids. Moreover, a protein particularly affected by GR is probably the orange carotenoid protein (OCP), which actually associates with the PBS and binds only keto-carotenoids (Wilson et al., 2006; Punginelli et al., 2009). Non-functional OCP would directly cause a “disturbance” of growth, as it plays a crucial role in photo-protection of the cells, via thermal energy dissipation, and in singlet oxygen quenching (Wilson et al., 2006; Stadnichuk et al., 2012). Consistent with the latter interpretation, the GR expressing ΔPSI strain (QC-GR) shows a phenotype of more readily bleaching cells, which may be caused by impaired exciton quenching in the PBS, and by consequence, a high concentration of ROS. It is relevant to note that the increased absorbance at ~485 nm is super-stoichiometric with the amount of expressed GR; i.e., the increased absorbance at 400–550 nm cannot be solely explained by carotenoids that could be bound to GR. An option to further resolve this would be to investigate the effect of expressing the GR mutants GR-G178F or GR-G178W, that cannot bind carotenoids, but maintain strong proton pumping (Balashov et al., 2010).

### The Challenges in Estimation of the Physiological Effect of GR Expression *in vivo*

The MIMS data indicate that both the PR-expressing strain (QC-PR) and the GR-expressing strain (QC-GR) show up to 50 % lower respiratory activity than the “empty plasmid” carrying strain (QC-0). This indicates that expression of PR and GR provides the ΔPSI strain with an extra pathway for proton motive force generation and/or ATP synthesis under proper illumination, thereby allowing these strains to come along with a lower rate of respiration than the control strain (QC-0). Nevertheless, only the PR-expressing strain (QC-PR) is able to convert more glucose into biomass rather than oxidize it for ATP production, and hence significantly stimulate growth, relative to the control strain. Presumably GR does contribute to energy conversion via extra proton motive force generation, but its expression may cause an energy deficit, possibly related to extra carotenoid biosynthesis, so that its physiological benefit roughly compensates the extra energy costs connected to expression of this proton pump (see Figure 5). Measurement of intracellular levels of adenine nucleotides could have been considered as an alternative assay of functional activity of the heterologously expressed pmf-generating proton pumps. However, as many feedback regulation mechanisms may operate in free energy transduction in cyanobacteria, the change in steady state ATP levels might be much smaller than the changes in rates of electron transfer that drive these changes in ATP level, up to the point that these feedback mechanisms might even cause an inverted relation between ATP-level (i.e., what one can measure) and the rate of ATP consumption (what actually matters).

Contrary to the widespread occurrence of proton pumping rhodopsins among microorganisms (Sabehi et al., 2005; Rusch et al., 2007; Atamna-Ismaeel et al., 2008; Campbell et al., 2008; Gonzalez et al., 2008; Koh et al., 2010; Boeuf et al., 2016), enhancement of growth and/or stress-survival mediated by such rhodopsins, has currently been identified only in a few natural hosts (Gómez-Consarnau et al., 2007, 2010; Kimura et al., 2011; Steindler et al., 2011; Wang et al., 2012; Akram et al., 2013; Feng et al., 2013; Palovaara et al., 2014). Given the great diversity of phylogenetic, genomic, and physiological backgrounds of natural proton-pumping rhodopsin-containing hosts, it would be no surprise if PR-based phototrophy might benefit its host via different mechanisms. Among those, promotion of starvation survival and stimulation of growth rate would be the two extremes, while some more subtle physiological or ecological benefits may still be too delicate to explore. Furthermore, the interactions between a rhodopsin, i.e., GR, and other energy-transducing systems could make this task very challenging.

Notably, additional characteristics of a proton pumping rhodopsin, such as its oligomeric state, binding of ligands like carotenoids, its pH-dependent pumping activity, as well as its voltage- and delta-pH dependent vectoriality (Lörinczi et al., 2009; Vogt et al., 2013; Choi et al., 2014), also have influence on its physiological efficacy. As GR distinctly differs from PR in several characteristics (e.g., oligomeric state, carotenoid binding, counterion pKa (Lörinczi et al., 2009; Balashov et al., 2010; Vogt et al., 2013; Choi et al., 2014; Chen et al., 2017; Jana et al., 2017) those differences could also contribute to GR not being able to enhance the growth rate of the ΔPSI strain of *Synechocystis*.

## Data Availability

All datasets generated for this study are included in the manuscript and/ or the supplementary files.

## Author Contributions

QC, FB, and KH designed the experiments. QC, JA, JS, and OC performed the experiments. QC and KH wrote the paper. SG, CF, and WdG contributed to the writing of the paper and the overall experimental design.

### Conflict of Interest Statement

KH is scientific advisor to the start-up company Photanol BV. This does not create a conflict of interest nor does it alter the authors' adherence to accepted policies on sharing data and materials. The remaining authors declare that the research was conducted in the absence of any commercial or financial relationships that could be construed as a potential conflict of interest.

## Abbreviations

RuBisCO, Ribulose-1: 5-bisphosphate carboxylase/oxygenase
Chl *d*: chlorophyll *d*
Chl *a*: chlorophyll *a*
Chl *f*: chlorophyll *f*
ΔPSI strain: PSI deletion strain of *Synechocystis*
WT: wild type *Synechocystis*
PR: proteorhodopsin
PR DNFS: Proteorhodopsin-D212N/F234S
Pipps, Piperazine-N: N′-*bis*(3-propanesulfonic Acid)
GR: *Gloeobacter* rhodopsin
MMAR, 3-methylamino-16-nor-1, 2, 3: 4-didehydroretinal
CFU: colony forming unit
MIMS: Membrane-inlet mass spectrometry
PMF: proton motive force
PAR: photosynthetically active radiation.
